# Isolated unilateral adrenal gland hemorrhage following motor vehicle collision: a case report and review of the literature

**DOI:** 10.1186/s13256-017-1506-x

**Published:** 2017-12-26

**Authors:** Anna Lehrberg, Bilal Kharbutli

**Affiliations:** Department of General Surgery, Henry Ford Wyandotte Hospital, 2333 Biddle Ave, Wyandotte, MI 48192 USA

**Keywords:** Adrenal, Blunt, Trauma, Hemorrhage

## Abstract

**Background:**

Adrenal gland trauma is a rare condition that typically stems from blunt force trauma, and is associated with multiple organ injuries. Alternatively, isolated adrenal gland trauma is extremely rare, accounting for only 1.5 to 4% of all adrenal trauma cases. While isolated adrenal trauma is a mostly self-limiting condition, it is potentially life-threatening, representing a significant cause of bleeding, and/or hypotension due to adrenal insufficiency and adrenal crisis. Due to its rare occurrence, there are no reported guidelines for monitoring and observing isolated adrenal trauma.

**Case presentation:**

Here we report on an isolated adrenal hemorrhage from a blunt trauma without associated injuries. A 53-year-old white man presented with abdominal pain after a high-speed motor vehicle accident. An initial evaluation revealed minimal abdominal pain and negative focused assessment with sonography for trauma examination; computed tomography imaging revealed a significant fluid collection consistent with adrenal hemorrhage. He was observed in our intensive care unit for 24 hours, and had stable hemoglobin and vital signs, after which he was discharged. At 1-month follow-up, he reported persistent intermittent abdominal pain, which was completely resolved by the 4-month follow-up.

**Conclusions:**

This case report demonstrates isolated adrenal gland injury resulting from significant blunt trauma to the abdomen. There are no current guidelines for monitoring isolated adrenal hemorrhage. Recognizing possible adrenal injury in blunt trauma cases is important due to potentially severe adrenal hemorrhage; therefore, we recommend follow-up with serial abdominal computed tomography until the resolution of hemorrhage and symptoms.

## Background

Adrenal gland hemorrhage can result from traumatic or non-traumatic etiologies. Non-traumatic adrenal gland hemorrhage is described in the literature occurring in the settings of antiphospholipid antibody syndrome, in heparin-associated thrombocytopenia and coagulopathies, or in the setting of severe physical stress or illness and multiorgan failure. Most of these are bilateral. Other potential causes of unilateral adrenal hemorrhage include primary adrenal or metastatic tumors.

Adrenal gland trauma (AGT) is a rare and underreported injury with an incidence rate that ranges from 0.03 to 4.95% of all trauma cases [1–3]. AGT is often a result of blunt trauma; it most commonly occurs in high impact injuries, such as motor vehicle collisions, and possesses a mortality rate that ranges from 7 to 32.6% [1]. Autopsy studies have reported a higher incidence of adrenal trauma (7.8 to 26% of trauma patients) than the incidence reported by emergency and trauma databases, suggesting that cases of AGT may go unreported [4, 5]. The underreporting of AGT may be due to the fact that it is coincidentally diagnosed in conjunction with the treatment of other life-threatening traumatic injuries of which the severity can distract from its diagnosis and treatment [2].

AGT is reported most commonly with associated injuries to ribs, thorax, liver, vertebrae, kidney, and spleen [1]. Consequences of AGT include acute or delayed hemorrhage that is potentially life-threatening. A retrospective analysis of trauma patient mortality revealed a significantly higher mortality of patients with AGT (32.6%) versus patients without AGT (7.1%) [6]. Most acute traumatic adrenal injuries were not isolated and were associated with other injuries including thoracic and rib injuries (>50%) and liver injury (> 40%) [1, 2]. The right adrenal gland is most commonly affected.

While isolated AGT is normally a benign, self-limiting condition that does not usually require surgery, adrenal hemorrhage is often associated with high injury severity of other intraabdominal organs and can be masked by other injuries [7]. There have been rare cases of endocrine-related syndromes after adrenal trauma with adrenal crisis and post-traumatic pheochromocytoma-like syndrome [8, 9].

Examples of isolated AGT without concomitant injury are extremely rare. Isolated adrenal injury has a reported incidence of only 1.5 to 4% of all adrenal traumas and less than 0.007% of the total traumas [1, 10, 11]. Here we report a case of isolated unilateral AGT and hemorrhage following blunt trauma resulting from a motor vehicle collision.

## Case presentation

A 53-year-old white man presented to our emergency department for blunt trauma following a high-speed motor vehicle collision as a restrained driver (he was wearing a three-point seatbelt). His history included nephrolithiasis 3 years ago that resolved with conservative medical therapy. He denied any surgical history. He was not on any anticoagulant or antiplatelet therapy prior to trauma. His social history included daily tobacco use and negative for any drug use. A family history did not reveal any coagulation disorders. On presentation, he complained of mild right upper quadrant and right flank pain. An examination revealed mild tenderness to deep palpation in his right upper quadrant and right flank, without evidence of ecchymosis, hematoma, or lacerations on his abdomen or his chest. There were no other abnormal findings on physical examination or laboratory values (Tables 1, 2, 3, 4, 5 and 6). He had normal vital signs without evidence of hypotension or tachycardia: blood pressure 158/110 mmHg, pulse 86 beats/minute, and temperature 37 °C (98.6 °F).

**Table 1:** Complete blood count with differential

	Reference range and units	Patient’s admission labs
WBC count	3.8–10.6 K/uL	6.6
RBC count	4.40–6.00 M/uL	4.74
Hemoglobin	13.5–17.0 g/dL	15.1
Hematocrit	41–53%	44.4
MCV	80–100 fl	93.6
MCH	26–34 pg	31.8
MCHC	31–37 g/dL	33.9
RDW	< 14.5%	13.1
Platelet count	150–450 K/uL	219
Neutrophil,%	%	57
Lymphocyte,%	%	33
Monocyte,%	%	7
Eosinophil,%	%	2
Basophil,%	%	1
Neutrophil, absolute	1.80–7.70 K/uL	3.80
Lymphocytes absolute	1.10–4.00 K/uL	2.10
Monocytes, absolute	0.00–0.80 K/uL	0.50
Eosinophils, absolute	0.00–0.70 K/uL	0.20
Basophils, absolute	0.00–0.20 K/uL	0.00

*MCH* mean corpuscular hemoglobin, *MCHC* mean corpuscular hemoglobin concentration, *MCV* mean corpuscular volume *RBC* red blood cells, *RDW* random distribution of red cell width, *WBC* white blood cells

**Table 2:** Comprehensive metabolic panel

	Reference range and units	Patient’s admission labs
Sodium	135–145 mmol/L	*133 (L)*
Potassium	3.5–5.0 mmol/L	3.6
Chloride	98–111 mmol/L	101
Carbon dioxide	21–35 mmol/L	26
Anion gap	3–13	6
Blood urea nitrogen	10–25 mg/dL	19
Creatinine	< 1.13 mg/dL	*1.24 (H)*
Comments: IDMS standardized		
Glucose	50–140 mg/dL	*150 (H)*
Comments: If fasting, glucose reference range = 70 to 100 mg/dL		
Calcium	8.2–10.2 mg/dL	8.5
GFR non-African American	> 60 ml/minute/1.73 m^2^	66

*GFR* glomerular filtration rate, *H* high, *IDMS* isotope dilution mass spectrometry, *L* low

**Table 3:** Hemoglobin and hematocrit trend

	Reference range and units	Admission	5 hours	8 hours	15 hours
Hemoglobin	13.5–17.0 g/dL	15.1	14.8	14.5	14.3
Hematocrit	41–53%	44.4	42.9	42.7	42.1

**Table 4:** Liver function test on admission

	Reference range and units	Patient’s admission labs
AST/SGOT	< 35 IU/L	*54 (H)*
ALT/SGPT	< 40 IU/L	*45 (H)*
Protein, total, serum	6.0–8.3 g/dL	7.0
Albumin	3.7–4.8 g/dL	4.2
Bilirubin, total	< 1.2 mg/dL	0.6
Bilirubin, direct	0–0.3 mg/dL	0.1
Alkaline phosphatase	0–120 IU/L	57
Globulin	2.5–4.1 g/dL	2.8
A/G ratio	0.9–1.8	1.5

*A/G* albumin to globulin ratio, *ALT* alanine aminotransferase, *AST* aspartate aminotransferase, *H* high, *SGOT* serum glutamic oxaloacetic transaminase, *SGPT* serum glutamic pyruvic transaminase

**Table 5:** Coagulation studies

	Reference range and units	Patient’s admission labs
Prothrombin time	12.1–14.5 seconds	13.0
INR		0.99
Comments: Usual intensity therapeutic range 2.0 to 3.0, high intensity therapeutic range 2.5 to 3.5, common critical alarm value 5.0		
PTT	22–36 seconds	25

*INR* international normalized ratio *PTT* partial thromboplastin time

**Table 6:** Urinalysis

	Reference range and units	Patient's UA
Clarity		Clear
Specific gravity	1.005–1.030	1.018
Urine pH	5.0–7.5	7.0
Protein	mg/dL	Negative
Glucose UA	mg/dL	Negative
Ketones	mg/dL	Negative
Bilirubin		Negative
Blood		*Trace (A)*
Urobilinogen	< 2.0 U/dL	< 2.0
Nitrite		Negative
Leukocyte esterase		Negative

*A* abnormal, *UA* urinalysis

**Fig. 1:**
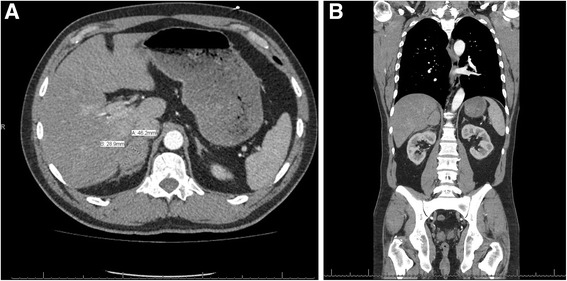
**a** Initial computed tomography scan at admission: right adrenal gland with an ovoid collection fluid consistent with blood measuring 4.6 × 2.9 cm in size with periadrenal stranding and with blood tracking along the inferior margin of the right hepatic lobe. **b** Coronal view of initial computed tomography at admission

A focused assessment with sonography for trauma (FAST) examination was performed in the emergency department and found to be negative, but owing to the high speeds involved in the crash, a computed tomography (CT) scan was subsequently ordered. The CT scan of his chest, abdomen, and pelvis revealed acute hemorrhage seen in the expected location of his right adrenal gland with an ovoid collection of increased density measuring 4.6 × 2.9 cm in size with periadrenal stranding and with blood tracking along the inferior margin of the right hepatic lobe (Fig. 1a). A normal right adrenal gland was not visualized due to the suspected hemorrhage in the area. The fluid seen on CT was consistent with blood product as opposed to adrenal mass. No other injuries were identified, specifically no injuries to his liver or kidneys. No rib or spine fractures were present.

He was admitted to our hospital for clinical observation and hemodynamic monitoring. His hemoglobin remained stable over 24 hours (Table 3). He had normal coagulation studies, as well as normal liver functions (Tables 4 and 5). He had slight elevation in his renal function with admission blood urea nitrogen (BUN) of 19 and creatinine (Cr) at 1.24. Repeat laboratory tests at 24 hours were normalized with BUN 14 and Cr 0.89. His urine analysis (UA) was clinically insignificant with trace amount of blood with 3/high-power field (HPF), rare bacteria, and it was negative for bilirubin, leukocyte esterase, and nitrites. A prior UA done in 2013 showed similar trace blood in urine secondary to left ureterolithiasis. He had no electrolyte abnormalities and had a stable 24 hours of vital signs; therefore, there was no clinical indication to continue further workup for possible adrenal dysfunction.

His clinical condition was stable with improvement in right upper quadrant and flank pain and tenderness. He was discharged from our hospital with restrictions on physical activities and he was asked to avoid anticoagulants. A follow-up repeat CT scan was scheduled to assess resolution of adrenal hematoma. Furthermore, because adrenal gland injury is not usually present in the absence of other injuries and his right adrenal gland was not visualized, occult neoplasm must be included in the differential diagnosis and required a follow-up.

**Fig. 2:**
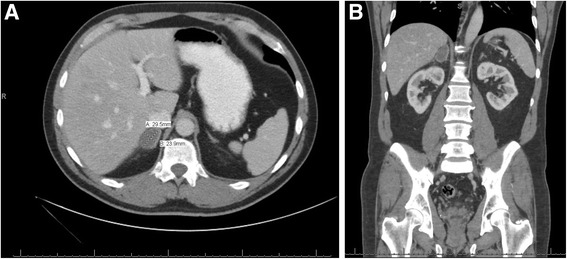
**a** One-month follow-up computed tomography scan. The right adrenal gland hemorrhage had improved and reduced to 3.0 × 2.4 cm in diameter. The previously noted right periadrenal fatty stranding was almost completely resolved. **b** Coronal view of 1-month follow-up

At the 1-month follow-up a CT scan with intravenously and orally administered contrast for better delineation of surrounding structures was completed and showed the adrenal gland hemorrhage had improved and reduced to approximately 3.0 × 2.4 cm in diameter, and the previously noted right periadrenal fatty stranding was mostly resolved (Fig. 2a and b). Again, there were no other abnormal findings noted. During the 1-month clinical examination, he reported intermittent right upper quadrant pain that occurred approximately one to two times per week but he was back to his regular activities. A physical examination showed no abdominal or flank tenderness.

**Fig. 3:**
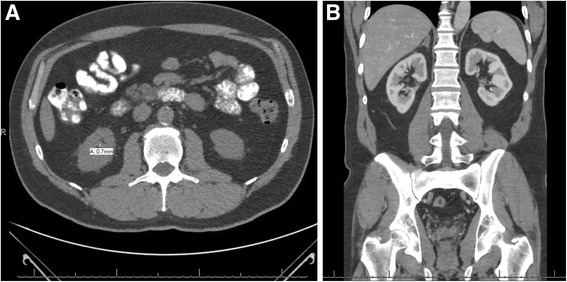
**a** Four-month follow-up computed tomography scan. Improving appearance of right adrenal gland with fluid measuring maximally 1.2 cm transverse diameter and no other identifiable lesions. Periadrenal fatty stranding was completely resolved. **b** Coronal view at 4-month follow-up

Due to persistent symptoms, he was instructed to follow-up in 3 months for another CT scan and examination. A 4-month CT scan was performed, which showed an improving appearance of right adrenal gland measuring maximally 1.2 cm transverse diameter and no other identifiable lesions (Fig. 3a and b). The time-dependent decrease in size of the right adrenal gland abnormality was consistent with hemorrhage, as opposed to adrenal neoplasm. He was asymptomatic at this time with resolution of abdominal pain and no symptoms of adrenal insufficiency.

## Discussion

The case of isolated adrenal gland injury presented here was unusual in that it involved a vehicle crash at high speeds, albeit without other injuries [3]. The presented case is not a new presentation but raises the question of how to follow hemorrhage of the adrenal gland without evidence of other organ injuries. Adrenal gland injuries are usually associated with other life-threatening injuries [1, 2, 12]. Accordingly, cases of isolated traumatic unilateral adrenal gland injuries are rare [3, 13–15].

The AGT injury presented here shares a number of similarities with the few previously reported cases of isolated unilateral AGT [3, 13–15]. Like most other cases of AGT, the major complaint in this case was abdomen pain in the right upper quadrant [3, 13–15]. However, an unusual aspect of this case that differentiates it from other unilateral adrenal injuries is that the pain was mild; a failure to report the severity of pain may result in underreporting of this type of injury. Other reported isolated unilateral adrenal injuries presenting with severe pain were the result of sports injuries [3, 15].

In addition, the AGT observed here is similar to the other cases reporting similar injury with regard to its being unilateral and right sided [3, 7, 13, 15]. In a retrospective analysis of trauma patients, Mehrazin *et al*. found that a higher number of patients incurred right-sided adrenal gland injuries (that is, 78.5%); the authors hypothesized that this higher number of right-sided adrenal gland injuries could be explained by two potential mechanisms: compression of the adrenal gland by surrounding organs or the physiology of the right adrenal gland itself [7]. The higher frequency of right-sided trauma includes: acute rise in intra-adrenal venous pressure due to compression of the inferior vena cava (IVC) during impact; deceleration forces causing the small adrenal arterioles to shear; and crushing between the spine and surrounding organs [16].

Bilateral AGT can lead to life-threatening adrenal insufficiency or adrenal crisis, which without treatment can progress to cardiovascular collapse and severe sepsis [1, 2]. A high index of suspicion is required for hypotensive polytrauma patients with possible bilateral adrenal injuries [17]. Unlike bilateral AGT, unilateral AGT is normally a self-limited condition that can be managed with conservative treatment; however, due to the anatomic location, minor trauma to the adrenal gland can cause severe adrenal hemorrhage [6, 15, 18].

## Conclusions

We reported here a rare case of isolated unilateral adrenal injury with hemorrhage from blunt trauma. The presence of adrenal neoplasm presenting as possible adrenal hemorrhage is described [19–21]. The need to follow-up and to rule out an underlying adrenal neoplasm should be considered, due to possible hemorrhage into a pre-existing adrenal mass. Currently there are no guidelines for follow-up for an isolated traumatic adrenal hemorrhage and, if documented, resolution or absence of underlying neoplasm is required due to the rarity of these cases. The diagnostic challenge in this case is the necessary clinical and radiologic follow-up for isolated adrenal hemorrhage. We propose an interval CT follow-up at 1 to 4 months for large, > 4 cm, adrenal gland hemorrhage based on known likelihood of incidental adenomas < 4 cm, with typical radiologic findings, such as smooth and homogenous lesions, to have low risk of malignancy [22]. In further management, if adenoma cannot be ruled out, it may be beneficial to complete a biochemical analysis at the follow-up to rule out functioning adenomas.
